# Response of Neutrophils to Extracellular Haemoglobin and LTA in Human Blood System

**DOI:** 10.1016/j.ebiom.2015.01.003

**Published:** 2015-01-13

**Authors:** Sae-Kyung Lee, Suh Yee Goh, Yuan Qi Wong, Jeak Ling Ding

**Affiliations:** Department of Biological Sciences, NUS Graduate School for Integrative Sciences and Engineering (NGS), National University of Singapore, 14 Science Drive 4, Singapore 117543, Singapore

**Keywords:** Haemolytic infection, Leukocytes, Extracellular haemoglobin, Lipoteichoic acid, Reactive oxygen species, *Staphylococcus aureus*, Toll like receptor

## Abstract

**Background:**

Haemolytic infection lyses red blood cells, releasing haemoglobin (Hb) into the plasma. Although recent studies showed that immune cells recognize redox-active cytotoxic extracellular Hb (metHb) bound to pathogen-associated molecular patterns (PAMPs), currently available information is limited to experiments performed in defined conditions using single cell lines. Therefore, a systemic approach targeting primary whole blood cells is required to better understand the cellular immune defence against metHb and PAMPs, when under a haemolytic infection.

**Methods:**

We investigated how human white blood cells, including neutrophils, respond to metHb and lipoteichoic acid (LTA) by measuring reactive oxygen species (ROS), signalling mediators (ERK and p38), NF-κB, cytokines, elastase secretion and cell activation markers.

**Findings:**

metHb activates NF-κB in TLR2-expressing HEK293 cells but not in normal or TLR9-expressing HEK293 cells. Treatment of isolated neutrophils with metHb increased production of ROS and expressions of IL-8, TNFα, and CD11b, which were further enhanced by metHb + LTA complex. While LTA stimulated the survival of neutrophils, it caused apoptotic cell death when complexed with metHb. The activation of neutrophils by metHb + LTA was subdued by the presence of other types of white blood cells.

**Interpretation:**

metHb and metHb + LTA complex are ligands of TLR2, inducing an unconventional TLR signalling pathway. Neutrophils are a highly sensitive cell type to metHb + LTA complex. During a haemolytic infection, white blood cells in the vicinity crosstalk to modulate neutrophil TLR-signalling induced by metHb and LTA.

## Highlights

• Extracellular haemoglobin (metHb) is a ligand for Toll-like receptor 2 (TLR2).
• metHb + lipoteichoic acid (LTA) complex synergistically activates neutrophils through an atypical TLR2 pathway.
• Neutrophils activated by metHb + LTA complex are modulated by other types of leukocytes.

## 1 Introduction

Extracellular haemoglobin (Hb) is readily oxidized into metHb, which is highly redox-active and cytotoxic due to its pseudoperoxidase activity (metHb-POX) (Jiang et al., 2007). Dangerous levels of cell-free metHb may persist in the plasma, for example in sickle cell anaemia (1.6 mg/ml) and paroxysmal nocturnal hemoglobinuria (5–20 mg/ml) (Schaer et al., 2013, Hartmann et al., 1966). The plasma metHb as well as pathogen-associated molecular patterns (PAMPs) are released into the plasma in an infection by haemolytic microbes, which may cause systemic inflammatory responses leading to multiple organ dysfunctions. metHb is normally rapidly scavenged by haptoglobin, scavenger receptor class (SR)-B1, and CD163. The internalized metHb undergoes detoxification and degradation (Subramanian et al., 2013, Schaer and Alayash, 2010). However, in severe haemolysis, massive levels of metHb overwhelm the capacity of metHb scavengers leading to excessive production of reactive oxygen species (ROS) by metHb-POX, which perturbs immune homeostasis (Olsson et al., 2012). metHb may bind to other damage-associated molecular patterns (DAMPs) and PAMPs, which are recognized by pattern recognition receptors such as TLRs in various immune cells, to trigger pro-inflammatory responses. When present in the plasma, metHb is a highly redox-reactive major DAMP that threatens the integrity of the white blood cells (WBCs), but its potentials to signal through TLRs are hitherto unclear (Lee and Ding, 2013). The methicillin-resistant strain of *Staphylococcus aureus* is a notorious haemolytic Gram-positive bacterium, which has become a major public health problem (Iwamoto et al., 2013, Stryjewski and Corey, 2014). Lipoteichoic acid (LTA) is the key immunostimulatory component of *S. aureus* that triggers TLR2-activating innate immune system of the host. Hb has been known to form a complex with *S. aureus* LTA to potentiate the immune stimulatory effect of LTA (Hasty et al., 2006). We previously reported the mechanism of ROS production by metHb-POX, showing that binding of LTA to metHb enhances the production of ROS, which not only kills the invading microbe, but is also harmful to the host blood cells (Jiang et al., 2007, Bahl et al., 2011, Du et al., 2010).

Neutrophils are one of the first responding immune cells to an infection. The migration of neutrophils into the site of infection–inflammation is mediated by PAMPs from microbes or DAMPs derived from disrupted host cells. At the site of infection, neutrophils rapidly combat pathogens by unleashing ROS and proteases. Furthermore, antimicrobial proteins stored in their cytoplasmic granules are released (Nathan, 2006). The azurophil granules contain myeloperoxidase, defensins, cathepsin G, and elastase, which are released upon activation and degranulation of the neutrophils. Amongst these, elastase which is a major serine protease is involved in various inflammatory responses (Pham, 2006). Neutrophils are known to interact with other leukocytes through cell–cell contact, and they secrete cytokines and chemokines. They modulate dendritic cell maturation and trafficking and are able to cross-present antigens to memory CD4^+^ T cells as well as to naïve CD8^+^ T cells, which subsequently amplify CD8^+^ T cell response to the antigen (Beauvillain et al., 2007). Direct interaction between neutrophils and T cells has been shown in the regression of cancer as well as infectious diseases (Stoppacciaro et al., 1993, Ma et al., 2009). ROS, produced by activated neutrophils, inhibits the effector functions of NK cells, while cytokines such as GM-CSF and IFN-γ released from activated NK cells, prolong the survival of neutrophils in an in vitro system (Costantini and Cassatella, 2011). Moreover, depletion of neutrophils impairs the recruitment of monocytes and lymphocytes to the inflammatory site. On the other hand, the immune suppressive capacity of neutrophils in T cell proliferation during acute systemic inflammation has also been reported (Pillay et al., 2012).

Considering the diverse functions of neutrophils in inflammation, we envisaged that neutrophils would play a significant role in response to plasma metHb during a haemolytic condition. Therefore, we investigated the response of neutrophils and the other types of blood cells, to metHb and LTA. Here, we show that metHb is an endogenous DAMP ligand for TLR2, and that neutrophils are one of the most sensitive cell types responding to (metHb + LTA)-induced production of ROS. Interestingly, this effect is diminished by the presence of other leukocytes, indicating that the white blood cells communicate with each other to modulate cellular response during a haemolytic infection.

## 2 Materials and Methods

All procedures followed the guidelines of the National University of Singapore Institutional Review Board (NUS-IRB Ref. code: B-14-063E). Buffy coats were obtained from the Blood Bank with appropriate informed consents from the volunteers.

### 2.1 Cells and Reagents

HEK293 clones expressing human TLR2, TLR9 and *S. aureus* LTA (LTA-SA) were purchased from InvivoGen (San Diego, California, USA). Human Hb, blasticidin, and hygromycin were from Sigma-Aldrich (St. Louis, MO, USA). Buffy coat from healthy donors was obtained from the Blood Bank, National University Hospital, Singapore. Primary blood cells were incubated in 5% CO_2_ at 37 °C in HEPES-buffered RPMI 1640 containing 100 U/ml penicillin, 100 μg/ml streptomycin, and 2% FBS. HEK293 cells were cultured in DMEM supplemented with 10% FBS, 100 U/ml penicillin, and 100 μg/ml streptomycin. Adult human Hb, which contains 96.5–98.5% HbA1 (α_2_β_2_ dimer) and 1.5–3.5% HbA2 (α_2_δ_2_ dimer), has been verified to be in the metHb state by spectrophotometric scanning between 500 and 700 nm (Du et al., 2010). As the endotoxin concentration in Hb was determined to be 2.86 EU/mg/ml by PyroGene Recombinant Factor C kit (Lonza Inc.), 1 mg/ml of Hb was pre-treated with 5 μg/ml of polymyxin B, which can scavenge 10 EU/ml of endotoxin. The metHb + LTA complex was pre-formed by co-incubating 1 mg/ml metHb with 10 μg/ml LTA for 30 min.

### 2.2 Isolation of Human Neutrophils and Cellular Assays

Buffy coats obtained from healthy donors were used for the isolation and enrichment of neutrophils with HetaSept^™^ (Stem Cell Technologies Inc.) according to the manufacturer's instructions. Immediately after isolation, the cells were incubated with different concentrations of metHb, LTA or metHb + LTA for 1 h (or as described in the figure legends), in RPMI medium followed by washing with PBS. For TLR2 blocking assay, 3.8 × 10^5^ neutrophils/well in 12-well plate were preincubated with TLR2 blocking antibody (20 μg/ml, eBioscience) for 1 h and then treated with metHb + LTA complex prepared as described above. For the assessment of intracellular ROS production, the cells were incubated with 1 μM CM-H_2_DCFDA for 30 min at 37 °C. After washing with PBS, the fluorescence signal of CM-H_2_DCFDA in the cells was measured by flow cytometry (CyAn ADP flow cytometer, Dako).

### 2.3 Isolation of Human Leukocytes and Stimulation

Human total leukocytes (referred as white blood cells, WBCs) were isolated from buffy coat using HetaSep^™^ followed by EasySep^™^ Human Positive Glycophorin A Depletion Cocktail (Stem Cell Technologies Inc.), according to the manufacturer's instructions. Human PBMCs were isolated from buffy coat by Ficoll-Paque density gradient centrifugation. The cells were incubated with metHb, LTA or metHb + LTA (as described above for neutrophils). Activated cells were assessed by flow cytometry for increased expression of activation markers, such as CD86, CD69, CD11b, LFA-1, DNAM-1, and Icam-1. ROS production induced in PBMCs or WBC treated with different stimulators was determined using CM-H_2_DCFDA as described above.

### 2.4 Chemiluminescence Assay for Haemoglobin Pseudoperoxidase (metHb-POX) Activity

The effect of PAMPs on the metHb-POX activity was assessed by measuring O_2_•^−^, using a chemiluminescence assay. O_2_•^−^-triggered chemiluminescence of Cypridina luciferin analog (CLA) was measured with a luminometer (Promega, Glomax 20/20), and expressed as relative luminescence units per second (RLU/s). metHb incubated with 25 μg/100 μl of each PAMP was added to a substrate mixture containing 20 μM CLA and 5 mM H_2_O_2_ in 100 μl PBS (pH 7.4) and the resulting chemiluminescence was immediately recorded in real-time up to 150 s, according to Jiang et al. (2007).

### 2.5 NF-κB Reporter Assay

Stably transfected HEK293/hTLR2-HA, HEK293/hTLR9, and HEK293/vector control cells were plated at the density of 2 × 10^5^/well in 24-well plates and transiently transfected for 24 h with NF-κB luc plasmid (Stratagene) or control plasmid (pRL-CMV, Promega) using Fermentas TurboFect. Transfected cells were treated with various concentrations of metHb, PAMPs or metHb + PAMPs for 6 h before luciferase LARII substrate (Promega) was added into the lysed cells. Stop & Glo reagent was added for *Renilla* luciferase reading. Luciferase activity was measured in relative light units of firefly fluorescence normalized against *Renilla* luciferase fluorescence units. The results were presented as relative luciferase activity against control treatment where the value from control vector-transfected cells was taken as 100%.

### 2.6 Western Blot Analysis

HEK293/hTLR2-HA cells were challenged with metHb (0.5 mg/ml), LTA (100 ng/ml) or metHb (0.5 mg/ml) + LTA (100 ng/ml) for 6 h. For cell lines, LTA was applied at the optimized concentration of 100 ng/ml. The cell lysates were analyzed by immunoblotting using antibodies against Phospho-p44/42 MAPK (Thr202/Tyr204), p44/42 MAPK (Erk1/2), Phospho-p38 MAPK (Thr180/Tyr182) and p38 MAPK (Cell Signaling). Beta-actin was detected as a loading control.

### 2.7 Flow Cytometry

Stimulated cells were incubated with Fcγ receptor blocking antibody for 20 min on ice and then stained for 30 min on ice with fluorophore-conjugated antibodies: CD56-APC, CD69-PEcy7, Icam-1-PE, CD86-Alexa Fluor 488, CD11b-FITC, CD66b-APC and CD16-PE, from eBioscience and LFA1-PECy5, CD14-APC/Cy7 and DNAM-1-FITC from Biolegend. After washing thrice with PBS, the expression of surface molecules was measured by flow cytometry (Beckman CyAn and BD BioScience Fortessa) and data were analyzed with Flow Jo software and Summit software. The mean fluorescence intensity from specific antibodies was obtained by subtracting the values from control isotype antibodies. Lymphocytes, monocytes, and granulocytes, amongst the WBC population, were gated based on side scatter and forward scatter, which were not affected by stimulation (Supplementary Fig. 1).

### 2.8 Apoptosis Assay

Cell apoptosis and necrosis were determined using the annexin V-FITC apoptosis detection kit and 7-AAD staining solutions (eBioscience) according to the manufacturers' instructions. Briefly, primary neutrophils and WBCs were separately stimulated for 10 h with different concentrations of metHb (0.25, 0.5, 1 mg/ml) or LTA (10 μg/ml, a concentration optimized for primary cells) either singly or combined. The cells were then washed with PBS and binding buffer, and incubated with FITC-conjugated annexin V and 7-AAD for 15 min at room temperature. After washing, the cell suspension was immediately analyzed on an LSRFortessa.

### 2.9 Cytokine Determination

Cell culture supernatants obtained from 1 h-stimulated neutrophils, WBC, and PBMC cultures were obtained by centrifugation at 500 ×*g* for 5 min and stored at − 80 °C until tested. TNFα and IL-8 were measured using OptEIA™ (BD Biosciences, USA) according to the manufacturer's instructions. Secreted elastase was determined using PMN elastase ELISA kit (eBioscience).

### 2.10 Statistical Analysis

Statistical significances were analyzed using ANOVA, post-hoc Bonferroni's test, regression analysis and 2-tailed Student's *t*-test.

## 3 Results

### 3.1 Modulation of metHb-POX Activity by PAMPs

**Fig. 1:**
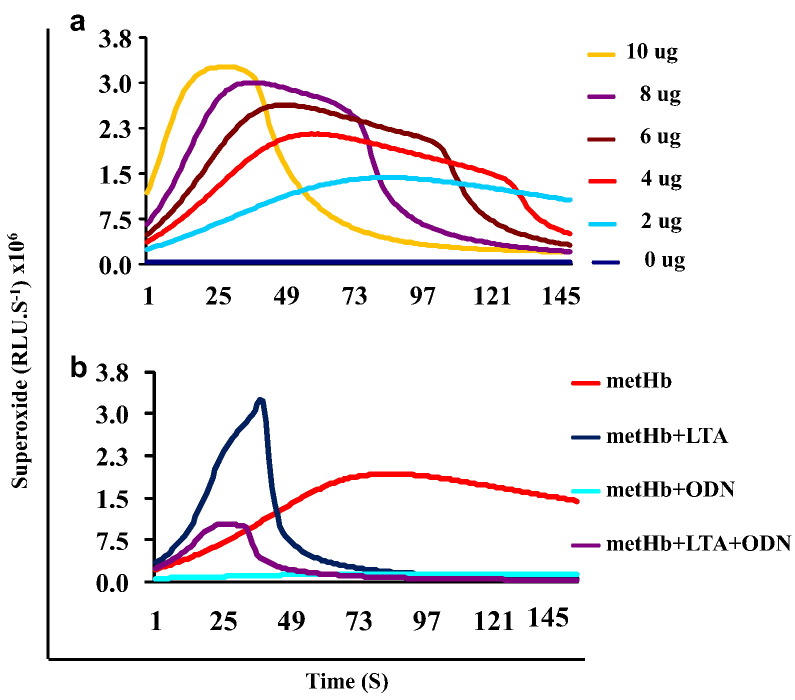
metHb-POX activity in the presence of LTA. (a) Kinetics of O_2_•^−^ production induced by different amounts of metHb. (b) Increase or inhibition of POX activity of metHb (4 μg/ml) by LTA and ODN2395.

metHb increased POX activity dose-dependently within the concentration range of 0–10 μg/100 μl as shown shortened reaction time (Fig. 1a). To assess the direct effects of various PAMPs on the metHb-POX activity, we incubated 4 μg/100 μl of metHb with 25 μg/100 μl of LTA or CpG DNA (ODN2395) for 30 min, and then determined the generation of O_2_•^−^ (ROS). LTA increased the metHb-POX activity while ODN decreased it (Fig. 1b). ODN2395 also reduced the metHb-POX activity induced by LTA. These results suggest that metHb-POX activity is directly modulated by specific PAMPs.

### 3.2 metHb Triggers Immune Response via TLR2

**Fig. 2:**
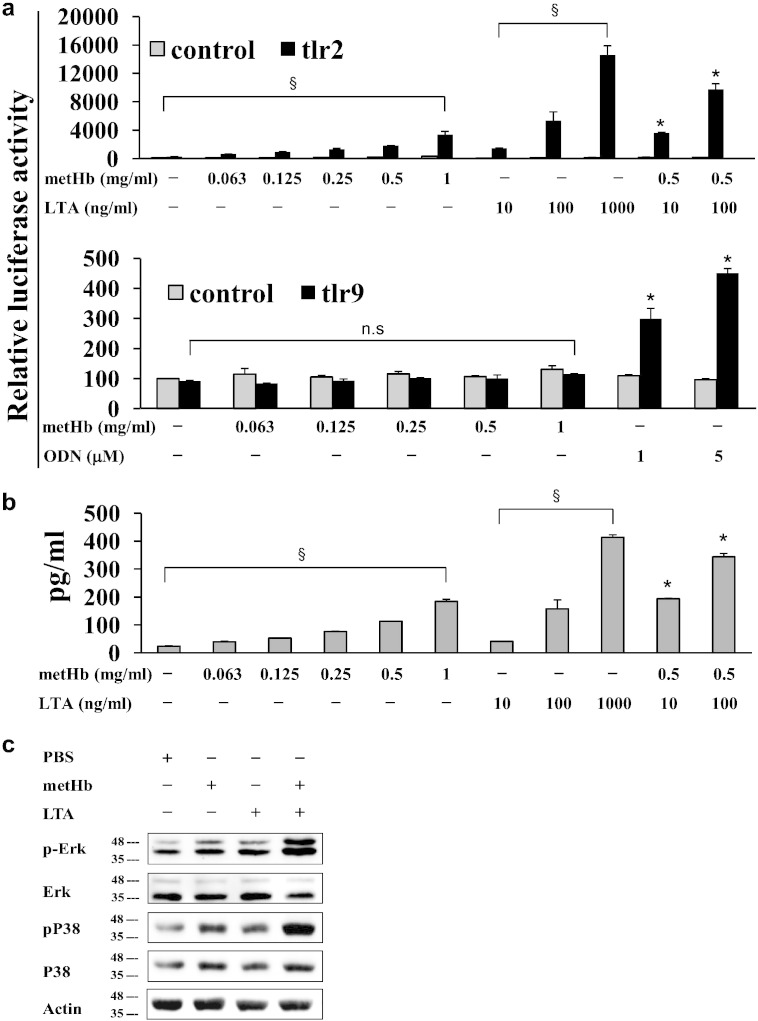
TLR2-mediated signal transduction by metHb, LTA and metHb + LTA. (a) NF-κB activation by metHb, LTA and metHb + LTA in HEK293 cells expressing hTLR2-HA and TLR9. (b) IL-8 secretion from HEK293 cells expressing hTLR2-HA induced by metHb, LTA or metHb + LTA complex. (c) Phosphorylation of ERK and p38 in HEK293 cells expressing hTLR2 treated with metHb, LTA or metHb + LTA complex. Data are representative of at least 3 independent experiments with similar results. §*P* < 0.05, dose dependency by regression analysis. * vs control, *P* < 0.05 by two-tailed Student's *t*-test.

Based on the above finding we hypothesized that certain TLR signal transduction may be modulated by metHb + TLR ligand complexes, exerting effects on the host innate immune response in haemolytic conditions. To test this hypothesis, we first assessed the effects of metHb on TLR2 and TLR9, which are both activated by *S. aureus* (Parker and Prince, 2012). Recombinant HEK293 cells which stably express hTLR2 or hTLR9 were co-transfected with NF-κB (the common mediator of TLR signalling) luciferase-expressing plasmid. The cells were treated 24 h later with metHb, LTA, ODN2395, or metHb + LTA and then NF-κB activation was measured by dual-luciferase reporter assay system. Interestingly, not only LTA but also metHb alone dose-dependently activated NF-κB in TLR2-expressing cells, while TLR9-expressing cells and control cells did not respond to metHb (Fig. 2a). These results, obtained by luciferase reporter assay, were further confirmed by IL-8 production and phosphorylation of ERK and p38 determined by ELISA and Western blotting, respectively (Fig. 2b, c). Altogether, these results suggest that metHb itself causes NF-κB activation through signals mediated by TLR2, leading to induction of chemokines such as IL-8.

### 3.3 Neutrophils are Highly Sensitive to metHb + LTA Complex

**Fig. 3:**
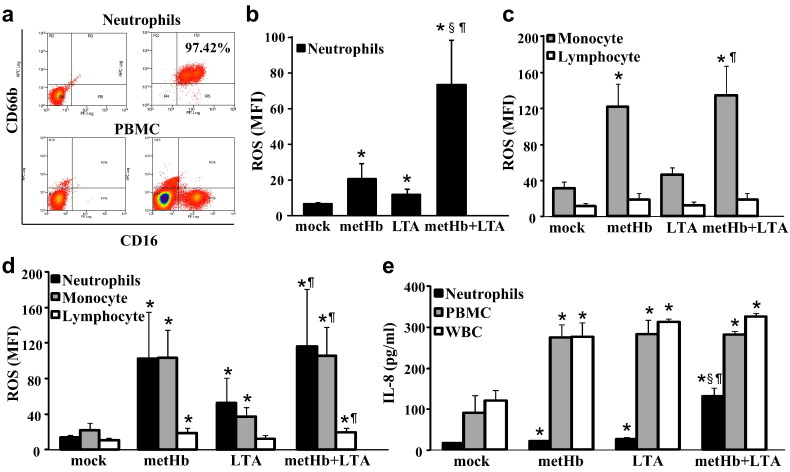
Intracellular ROS production and IL-8 secretion. (a) Purity of isolated neutrophils (CD66b^+^ CD16^+^) and PBMC was verified by flow cytometry. The CD16^+^ CD66b^−^ cell population in PBMC represents a mixture of natural killer cells and a minor sub-population of monocytes. Left, unstained; right, immunostained. ROS produced in (b) isolated neutrophils, (c) PBMC and (d) WBC. Different cell types in PBMC and WBC were identified by forward scatter and side scatter. (e) IL-8 secretion from isolated neutrophils, PBMC and WBC. Cells were stimulated with metHb (1 mg/ml), LTA (10 μg/ml) or metHb (1 mg/ml) + LTA (10 μg/ml) complex for 1 h. Mock was buffer only treatment. Data represent mean fluorescence intensity (MFI) ± SD of 3 independent experiments. * vs mock, § vs metHb, ¶ vs LTA, *P* < 0.05 by one-way ANOVA followed by post hoc Bonferroni's test.

To investigate the sensitivity of the different blood cell types to cell-free Hb and LTA, we prepared neutrophils, PBMC, and whole WBC separately (Fig. 3a). Both metHb and LTA increased ROS production in isolated neutrophils, which was further synergistically increased by treatment with metHb + LTA (Fig. 3b). Amongst the PBMC population, metHb induced more ROS production in monocytes than in lymphocytes, but metHb + LTA did not further induce more ROS (Fig. 3c). Consistently, we found that when WBCs were treated with metHb, the neutrophils and monocytes within the WBC population produced increased levels of ROS as compared to lymphocytes (Fig. 3d). However, when the neutrophils were analyzed in the presence of other WBCs, the metHb + LTA complex did not synergistically increase ROS production in neutrophils (Fig. 3d). IL-8 production from the isolated neutrophils was also further increased by metHb + LTA complex compared to metHb or LTA alone, while its increase in PBMC or WBC induced by metHb + LTA is not different from the level induced by metHb or LTA alone (Fig. 3e). Compared to neutrophils, PBMC treated with metHb produced much higher level of IL-8, while metHb + LTA complex did not further increase IL-8 in PBMC. These results suggest that neutrophils are highly sensitive to the imminent threat of metHb + LTA complex, which prevails in a haemolytic infection, for example by *S. aureus* and the stimulation of neutrophils by metHb + LTA is modulated by other WBCs.

### 3.4 Activation of Neutrophils by metHb + LTA is Mediated by TLR2 and Other Receptors

**Fig. 4:**
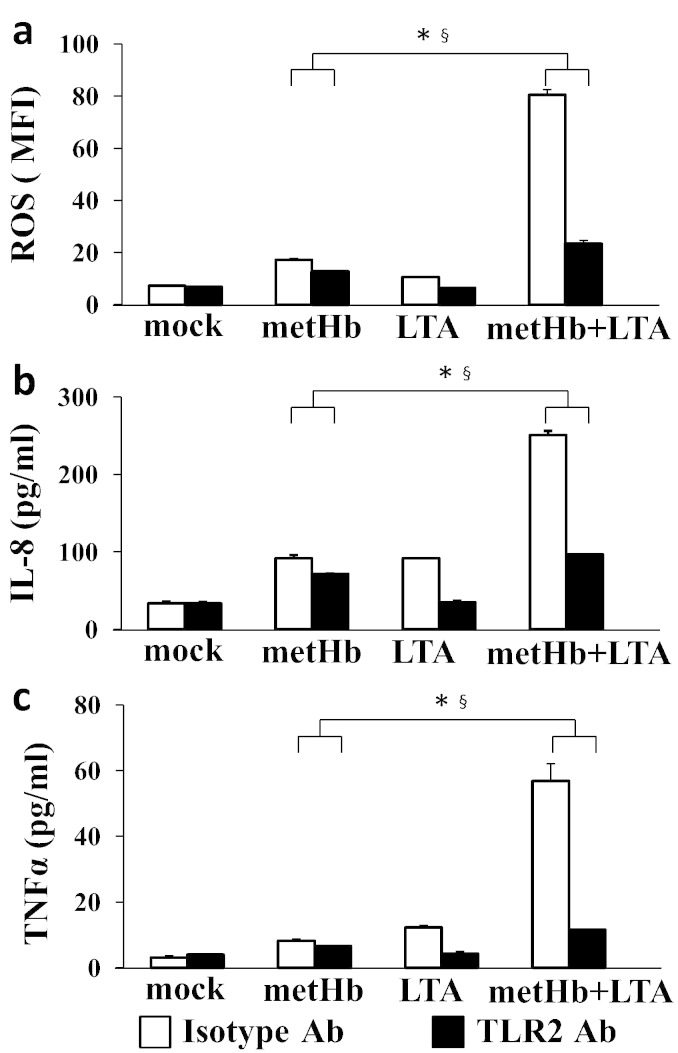
TLR2-dependent ROS production induced by metHb, LTA or metHb + LTA complex. Isolated neutrophils were pretreated with either TLR2 blocking Ab or isotype Ab for 1 h prior to the treatment with metHb (1 mg/ml), LTA (10 μg/ml) or metHb (1 mg/ml) + LTA (10 μg/ml) complex for 1 h. Mock was buffer only treatment. (a) Intracellular ROS determined using CM-H_2_DCFDA dye and measured by flow cytometry. (b) IL-8 secretion determined by ELISA. (c) TNFα secretion determined by ELISA. Data are representative of at least 3 independent experiments with similar results. * and § are effects of metHb + LTA combination and TLR2 antibody, respectively. *P* < 0.05 by two-factor ANOVA.

Human neutrophils have been shown to express all TLRs except TLR3 (Hayashi et al., 2003). Although it is well-known that LTA specifically targets TLR2, it is not certain whether during an *S. aureus* haemolytic infection, the extracellular metHb (with/without LTA) specifically signals via TLR2. To determine whether metHb alone or metHb + LTA complex induces immune signalling via TLR2, isolated neutrophils were separately pretreated with TLR2-blocking antibody or control isotype antibody. The synergistic increases of ROS, IL-8, and TNFα in isolated neutrophils induced by metHb + LTA were markedly decreased by treatment with TLR2-blocking antibody. Increases of ROS in neutrophils by metHb alone were also decreased by TLR2-blocking antibody, although the changes were not as significant as metHb + LTA treatment (Fig. 4). However, compared to mock control (buffer treatment), the levels of ROS and TNFα produced by metHb and metHb + LTA in the presence of TLR2-blocking antibody were significantly higher. This suggests that the metHb + LTA complex elicits synergistic activation of neutrophils both through TLR2-dependent and -independent pathways, indicating the potential involvement of other receptors besides TLR2.

### 3.5 metHb Controls the Survival/Death of Leukocytes

**Fig. 5:**
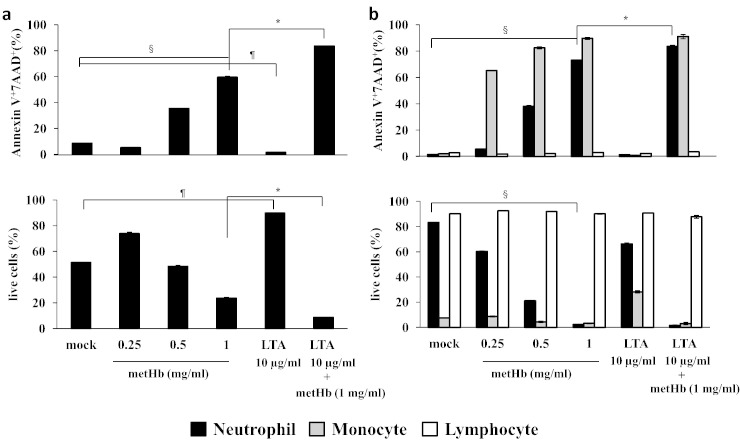
metHb-induced apoptosis. (a) isolated neutrophils, (b) WBC. The apoptosis was assessed by detecting Annexin V^+^/7AAD^+^ cells. The % of live cells was calculated with Annexin^−^/7AAD^−^ cell population. Mock was buffer only treatment. Data are means of duplicate experiments. §*P* < 0.05, dose dependency by regression analysis. *, ¶ *P* < 0.05 by two-tailed Student's *t*-test.

To ascertain whether metHb and LTA affect neutrophil survival or death, both isolated neutrophils and WBC were treated with metHb, LTA or metHb + LTA complex and the apoptosis of the cells was assessed by detecting Annexin V^+^/7AAD^+^ cells. metHb alone was found to induce apoptosis in neutrophils regardless of the presence of the other cell types (Fig. 5a, b). The metHb + LTA complex significantly enhanced the apoptotic effect. However, LTA itself did not induce apoptosis in either the neutrophils or the other leukocytes, but promoted the survival of neutrophils. While monocytes underwent cell death caused by metHb and metHb + LTA, the viability of the lymphocytes remained unaffected by these treatments (Fig. 5b). Taken together, these results indicate that extracellular cytotoxic metHb elicits different impacts on the death/survival of different leukocytes.

### 3.6 Neutrophils Express Elastase and Adhesion Molecules When Encountering metHb

**Fig. 6:**
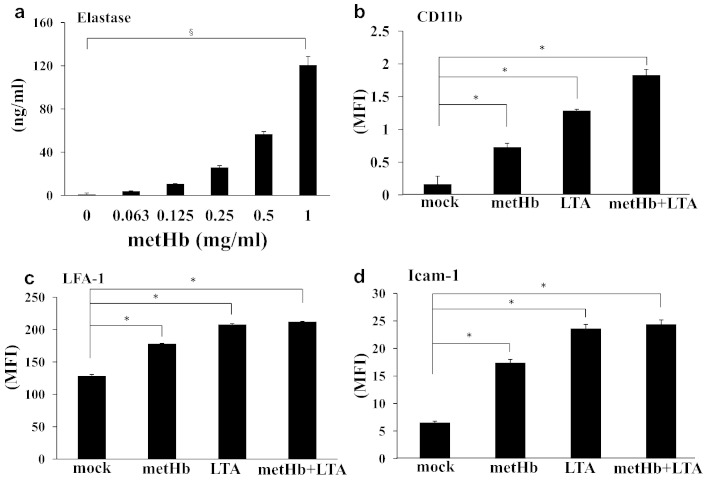
Activation of neutrophils by metHb. (a) Elastase secretion from metHb-stimulated neutrophils measured by ELISA. (b–d) Expression of CD11b, LFA-1, and Icam-1 on neutrophils. Mock was buffer only treatment. Data represent mean fluorescence intensity (MFI) ± SD. §*P* < 0.05, dose dependency by regression analysis. Data are means of duplicate experiments. *P < 0.05 by two-tailed Student's *t*-test.

Since elastase plays a major role in neutrophil extracellular trap formation, transvascular migration, and apoptosis (Oltmanns et al., 2005), we investigated the effects of metHb on elastase secretion from neutrophils. ELISA showed that metHb dose-dependently induced elastase secretion (Fig. 6a). Interestingly, treatment of neutrophils with metHb, LTA or metHb + LTA also increased the expression of adhesion molecules such as CD11b, LFA-1, and Icam-1 (Fig. 6b–d). The metHb- or (metHb + LTA)-mediated production of elastase and adhesion molecule in the neutrophils indicates that the migration of neutrophils to the sites of inflammation could be facilitated by metHb and LTA.

### 3.7 metHb Amplifies the Inflammatory Response Through Monocytes and T Cells

**Fig. 7:**
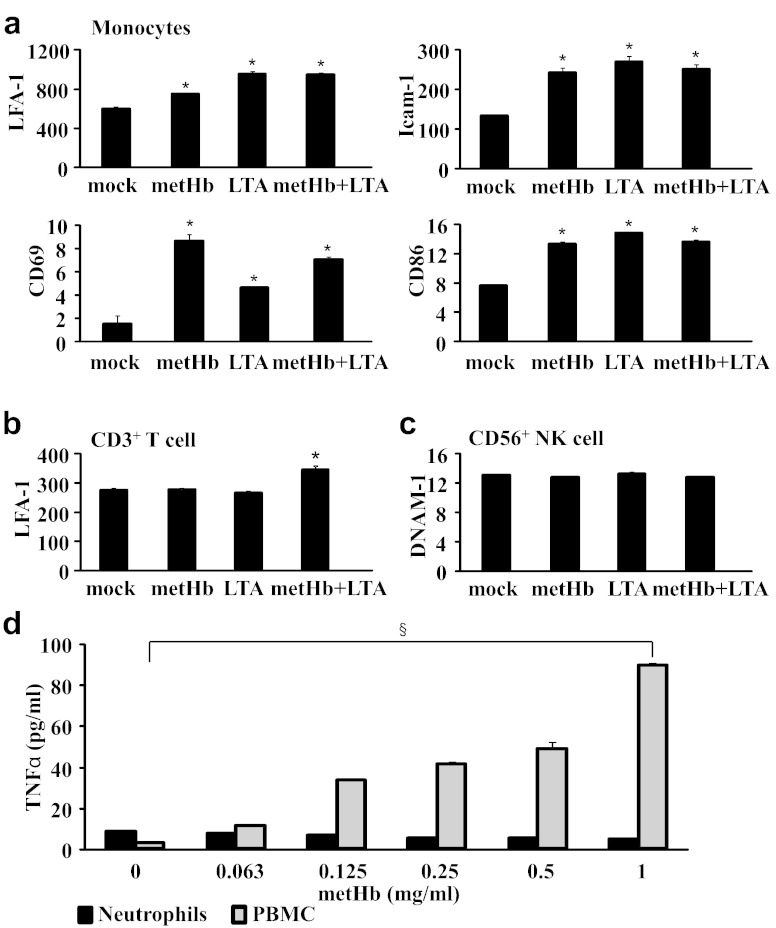
Expression of activation markers on human WBCs. Expression, in mean fluorescence intensity (MFI), of (a) LFA-1, Icam-1, CD69 and CD86 in monocyte, (b) LFA-1 in T cells and (c) DNAM-1 in NK cells. Isolated WBCs from buffy coat were treated with metHb (1 mg/ml), LTA (10 μg/ml) or metHb (1 mg/ml) + LTA (10 μg/ml) complex for 1 h and then cell surface markers, CD3 and CD56, were stained for the identification of T and NK cells by flow cytometry. Monocytes within WBCs were gated based on side scattering and forward scattering. Mock was buffer only treatment. (d) TNFα secretion from metHb-stimulated neutrophils and PBMC, measured by ELISA. Data are means of duplicate experiments. * vs mock *P* < 0.05, §*P* < 0.05, dose dependency by regression analysis.

As the infiltration of neutrophils into the infection–inflammation site is followed by recruitment of other immune cells, we then investigated whether metHb and LTA would activate the other WBCs besides neutrophils, which may consequently regulate the responses of neutrophils. To evaluate this possibility, we assessed PBMCs for the expression of diverse activation cell surface markers such as LFA-1, Icam-1, CD69, CD86, and DNAM-1 by flow cytometry, and quantified TNFα by ELISA. We found that monocytes respond to both metHb and LTA by up-regulating LFA-1, Icam-1, CD69, and CD86, although they were not further increased by metHb + LTA (Fig. 7a). However, LFA-1 marker in CD3^+^ T cells was increased by treatment with metHb + LTA relative to metHb or LTA only (Fig. 7b). DNAM-1 was consistently expressed on NK cells (CD56^+^) regardless of the treatments (Fig. 7c). Interestingly, TNFα expression in neutrophils was unchanged by metHb. In contrast, there was a dose-dependent increase in TNFα produced by the PBMCs (Fig. 7d).

On the premise that: (i) LFA-1 is required for T cell migration to target tissues as well as for optimal activation (Berlin-Rufenach et al., 1999), and (ii) proliferation of T cells and IFN-γ production from T cells are dependent on the expression of CD80 and CD86 in monocytes (Subauste et al., 1998), our results imply that metHb amplifies the inflammatory response in WBCs by stimulating not only neutrophils but also monocytes and T cells.

## 4 Discussion

One of the underlying mechanisms of the cytotoxicity of metHb is its ability to generate ROS and scavenge NO; the latter is known to play a protective role in vascular homeostasis. Recent studies in our lab have shown that the POX activity of metHb is triggered synergistically by microbial proteases and PAMPs such as LTA or LPS to produce ROS (O_2_•) which was confirmed in this study (Jiang et al., 2007). Binding of LTA or LPS to metHb causes a conformational change of metHb which may explain the increase in metHb-POX activity. But we found, in this study that ODN2395 dramatically reduced the POX activity of metHb as well as that of metHb + LTA complex (Fig. 1b). The decreased POX activity appears to be due to the direct binding of ODN2395 to metHb, which probably caused allosteric inhibition rather than a direct catalytic site competition against LTA. These results suggest differential modulation of the metHb-POX activity by different PAMPs during a haemolytic infection.

By further investigations on the effects of interaction between metHb and LTA on live human leukocytes, we discovered that metHb is an endogenous ligand for TLR2 (Fig. 2a). As confirmed in our experiment, a typical TLR2 activation by LTA has been known to inhibit apoptosis of neutrophils (Lotz et al., 2004). However, we observed metHb to induce apoptosis of neutrophils, which was significantly increased by metHb + LTA complex. As anticipated, blocking TLR2 in isolated neutrophils reduced the LTA-induced ROS production to basal level compared to mock control (buffer treatment). Interestingly, higher levels of ROS were produced by metHb and metHb + LTA compared to mock control, even in the presence of TLR2 antibody. These results suggest that there are other receptors on the neutrophils that may interact with metHb and metHb + LTA complex, besides TLR2. Since innate immune responses are mediated multifariously, it is conceivable that metHb-PAMPs modulate the functions of TLRs to stimulate distinct signalling pathways in diverse immune cells, which warrants future investigation at the systemic level. Supporting this is our earlier demonstration of the co-operation of monocytes with endothelial cells in the endocytosis of extracellular metHb^4^. Chronic haemolytic conditions, which prevail in sickle cell anaemia, are accompanied by systemic endothelial activation resulting in more adhesion of blood cells (Chen et al., 2011).

Here, our ex vivo systemic analysis of the impact of extracellular Hb and LTA on whole WBC populations demonstrates that neutrophils are as sensitive as monocytes to metHb. Interestingly other leukocytes in the whole blood system seemed to dampen the synergistic effect of metHb + LTA on the neutrophils. For example, the presence of other blood cell types suppressed further increase of ROS production induced by metHb + LTA compared to metHb in neutrophils; otherwise the metHb + LTA caused synergistic increase in ROS production in isolated neutrophils (Fig. 3b, d). Plausibly, the clearance of metHb by scavenging receptors such as CD163 and (SR)-B1, which are known to be present on macrophages and monocytes, partially relieves the impact of metHb + LTA on the neutrophils. However, metHb- or (metHb + LTA)-induced increase in the expressions of activation markers and cytokines in the other leukocytes indicates that the other leukocytes are more actively engaged in response to the effects of metHb on neutrophils. Particularly, the increase in cell adhesion molecules caused by metHb, LTA or metHb + LTA, in various types of leukocytes suggests that upon encountering metHb and LTA (e.g. in a Gram-positive haemolytic infection), the neutrophils probably interact with other leukocytes through cell–cell contact, and they secrete cytokines and chemokines leading to the modulation of effector cell function at a focal site of inflammation (Fig. 6, Fig. 7).

The regulation of neutrophil survival and death is critical to resolve inflammation efficiently. TLR agonists and inflammatory cytokines have been reported to delay apoptosis of neutrophils, which is associated with the expression of anti-apoptotic molecules such as survivin protein and myeloid leukaemia cell differentiation protein (Francois et al., 2005, Baumann et al., 2003, Altznauer et al., 2004). Furthermore, activated NK cells and T cells drive IFNγ and GM-CSF to prolong the human neutrophil survival (Costantini et al., 2010, Pelletier et al., 2010). We found that metHb does not increase TNFα production in isolated neutrophils (Fig. 4), whereas metHb + LTA does. However, metHb induces TNFα in whole WBC, resulting in more severe apoptosis of neutrophils compared to the effect in isolated neutrophils (Fig. 5). It was reported that low concentrations of TNFα enhance the survival of neutrophils, but high concentrations induce apoptosis (Cross et al., 2008). Our results suggest that apoptosis of neutrophils induced by metHb and metHb + LTA, when in the presence of other leukocytes, may be caused by both: (i) TNFα produced from the other types of leukocytes and (ii) ROS produced in the neutrophils.

**Fig. 8:**
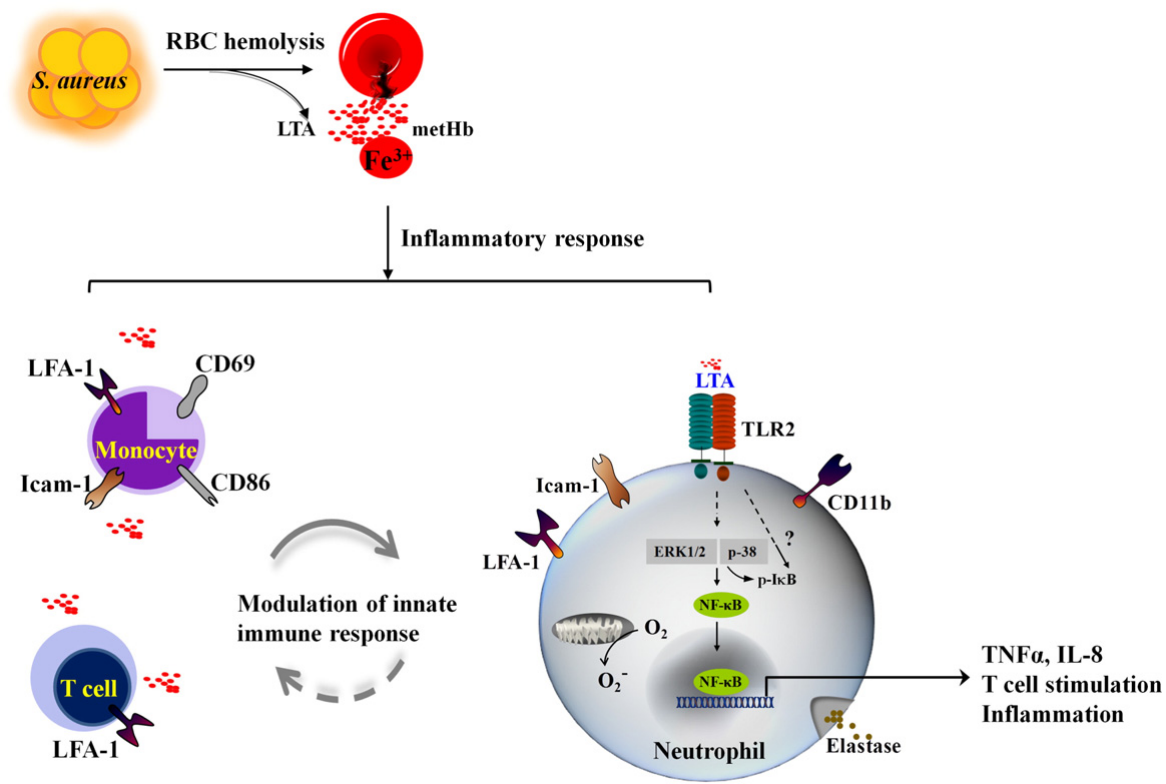
Schematic diagram of WBC response to metHb + LTA. Haemolytic bacterial infection e.g. by *S. aureus*, releases Fe^3 +^ metHb into the plasma, which is highly redox-active and interacts with LTA. Neutrophils respond highly sensitively to metHb + LTA complexes by activating unconventional TLR2-mediated signal transduction. Amongst the other leukocytes, monocytes and T cells are activated by metHb, LTA and metHb + LTA by expressing adhesion and costimulatory molecules leading to modulation of neutrophil's responses. The detailed molecular mechanism (denoted as “?” on dashed arrows) of TLR2 signal transduction induced by Hb + TLR ligand complex remains to be elucidated.

Overall, our findings provide mechanistic insights on how metHb, together with LTA, released during a systemic Gram-positive haemolytic infection triggers neutrophil response and how the other leukocytes might collaborate to orchestrate cellular defence as illustrated in Fig. 8. Future characterization of the proposed unconventional TLR pathway could provide deeper understanding on how our blood system circumvents the danger of the redox-active metHb, and PAMPs to restore homeostasis.

The following are the supplementary data related to this article.

Supplementary Fig. 1 White blood cells treated for an hour with mock treatment PBS (panels a, e, i, m); metHb-only (panels b, f, j, n); LTA-only (panels c, g, k, o); and a combination of metHb with LTA (panels d, h, l, p), were stained with cell markers: CD66b, CD16 and CD14. CD66b^+^/CD16^+^ cells were designated granulocytes (Q2 of panels e, f, g and h), and CD66b^−^/CD16^−^ cells (Q4) were further gated for CD14. CD66b^−^/CD16^−^/CD14^+^ cells were designated monocytes and CD66b^−^/CD16^−^/CD14^−^ cells were designated lymphocytes (panels i, j, k, l). These populations were back-gated and their FSC–SSC plots are represented (panels m, n, o, p). There was no significant change in the FSC–SSC profiles of granulocytes (red), monocytes (green) and lymphocytes (black) of immuno-labelled WBC after treatment.

## Declaration of Interests

The authors declare no competing financial interests.

## Author Contributions

S.K.L., S.Y.G. and Y.Q.W. performed the experiments. S.K.L. and J.L.D. conceived and designed the study. S.K.L., S,Y.G. and J.L.D. analyzed the data and wrote the manuscript.

## Funding

This study was supported by the Ministry of Education, Singapore (grant: MOE2013-T2-2-007) and the NUS Graduate School for Integrative Sciences and Engineering (NGS Grant reference C-154-000-017-091). The funding bodies do not play any role in the study design, data collection, data analysis, interpretation or writing of the manuscript.
