# Supplementary material for: Response of Neutrophils to Extracellular Haemoglobin and LTA in Human Blood System
Source: eBioMedicine. 2015 Jan 13;2(3):225–33. doi: 10.1016/j.ebiom.2015.01.003 (PMC4485489; doi:10.1016/j.ebiom.2015.01.003)
Supplement: Supplementary Fig. 1 — White blood cells treated for an hour with mock treatment PBS (panels a, e, i, m); metHb-only (panels b, f, j, n); LTA-only (panels c, g, k, o); and a combination of metHb with LTA (panels d, h, l, p), were stained with cell markers: CD66b, CD16 and CD14. CD66b+/CD16+ cells were designated granulocytes (Q2 of panels e, f, g and h), and CD66b−/CD16− cells (Q4) were further gated for CD14. CD66b−/CD16−/CD14+ cells were designated monocytes and CD66b−/CD16−/CD14− cells were designated lymphocytes (panels i, j, k, l). These populations were back-gated and their FSC–SSC plots are represented (panels m, n, o, p). There was no significant change in the FSC–SSC profiles of granulocytes (red), monocytes (green) and lymphocytes (black) of immuno-labelled WBC after treatment. [file mmc1.doc]

**Response of neutrophils to extracellular haemoglobin and LTA in human blood system**

Sae-Kyung Lee, Suh Yee Goh, Yuan Qi Wong and Jeak Ling Ding





**Supplementary Figure 1.** White blood cells treated for an hour with mock treatment PBS (panels a, e, i, m); metHb-only (panels b, f, j, n); LTA-only (panels c, g, k, o); and a combination of metHb with LTA (panels d, h, l, p), were stained with cell markers: CD66b, CD16 and CD14. CD66b+/CD16+ cells were designated granulocytes (Q2 of panels e, f, g and h), and CD66b-/CD16- cells (Q4) were further gated for CD14. CD66b-/CD16-/CD14+ cells were designated monocytes and CD66b-/CD16-/CD14- cells were designated lymphocytes (panels i, j, k, l). These populations were back-gated and their FSC-SSC plots are represented (panels m, n, o, p). There was no significant change in the FSC-SSC profiles of granulocytes (red), monocytes (green) and lymphocytes (black) of immuno-labeled WBC after treatment.
